# The impact of *Helicobacter pylori* infection on low skeletal muscle mass risk in Chinese women over 40: a cross-sectional analysis

**DOI:** 10.3389/fcimb.2023.1289909

**Published:** 2024-01-03

**Authors:** Xiaohui Xu, Yidan Qian, Kejia Jin, Junpeng Chen, Jiayue Fu, Chengshui Chen, Zaisheng Zhu

**Affiliations:** ^1^ Department of Medical Care Center, The First Affiliated Hospital of Wenzhou Medical University, Wenzhou, China; ^2^ The First School of Medicine, School of Information and Engineering, Wenzhou Medical University, Wenzhou, China; ^3^ Key Laboratory of Interventional Pulmonology of Zhejiang Province, The First Affiliated Hospital of Wenzhou Medical University, Wenzhou, China; ^4^ Department of Pulmonary and Critical Care Medicine, Quzhou People’s Hospital, The Quzhou Affiliated Hospital of Wenzhou Medical University, Quzhou, China; ^5^ Department of Pulmonary and Critical Care Medicine, The First Affiliated Hospital of Wenzhou Medical University, Wenzhou, China

**Keywords:** low skeletal muscle mass, 13C-urea breath test, *Helicobacter pylori* infection, risk factors, female

## Abstract

**Background:**

Sarcopenia can lead to significant personal, social, and economic burdens. The diagnosis of sarcopenia heavily relies on the identification of Low Skeletal Muscle Mass (LSMM), which is an independent predictor of frailty, disability, and increased risk of death among seniors. Women have physiologically lower levels of skeletal muscle mass than men, and female sarcopenia appears to be more influenced by menopause. They also tend to have higher body fat levels than man, which increases the risk of sarcopenia obesity. On another front, it’s also recognized that humans are largely prone to *Helicobacter pylori (H. pylori)* infection, with global prevalence rates often surpassing 50%. Nevertheless, the interconnection between *H. pylori* infection and LSMM remains relatively unexplored. Hence, our study specifically targeted women as the research population and sought to explore several risk factors for LSMM. Additionally, we delved into the potential correlation between LSMM and *H. pylori* infection in women, hoping to gain insights into potential preventative measures or treatment options that may enhance the quality of life for women affected by sarcopenia.

**Methods:**

We conducted a cross-sectional study among women aged over 18 years undergoing physical examination. We performed 13C-urea breath test (UBT) for diagnosis of *H. pylori* infection and Bioelectrical impedance analysis (BIA) for the assessment of LSMM. Logistic regression models were used to analyze the associations of *H. pylori* infection with LSMM.

**Results:**

This study enrolled 1984 Chinese women who were undergoing health check-ups. A univariate logistic regression analysis did not reveal a direct correlation between *H. pylori* infection and LSMM among this female population (OR=1.149, 95% CI 0.904-1.459, *p*=0.257). Yet, upon dividing the participants into age-based subgroups, an evident link was observed between *H. pylori* infection and LSMM in women aged 40 or above (OR=1.381, 95%CI 1.032-1.848, *p*= 0.030). After adjusting for variables including Age, BMI, TP, ALK, Cre, this relationship remained statistically relevant (OR=1.514, 95%CI 1.085-2.113, *p*= 0.015).

**Conclusions:**

Women who are over 40 years old and currently infected with *H. pylori* have an increased risk of developing LSMM. Therefore, timely treatment for *H. pylori* eradication is recommended for this group of women to reduce the occurrence of LSMM.

## Introduction

Helicobacter pylori (*H. pylori*) is a gram-negative, curved bacillus that was discovered and identified by Marshall and Warren in 1983 from gastric mucosa (Marshall and Warren, 1984). Studies have confirmed that *H. pylori* has coexisted with humans for 58,000 years (Linz et al., 2007), and can be found in approximately half of the world’s population with infection levels reaching over 70% in developing countries (Bravo et al., 2018). And The analysis of 410,879 participants from 73 countries showed that the global prevalence of *H. pylori* infection in women was 42.7% (Zamani et al., 2018). The association between *H. pylori* and conditions such as gastric cancer, peptic ulcer, and gastric lymphoma has been widely acknowledged (Hunt et al., 2011). However, recent studies have revealed a growing correlation between *H. pylori* and various extra-gastric disorders (Franceschi and Gasbarrini, 2007). These include a positive association with non-alcoholic fatty liver and adults metabolic syndrome (Chen et al., 2015; Buzás, 2020), as well as an inverse association with asthma, reflux disease, and allergic disorders (Cover and Blaser, 2009).

Identified as a muscular disorder in the International Classification of Disease (ICD-10: M62) (Lj and Mo, 2017), sarcopenia is typically distinguished by decreased muscle mass, reduced muscular strength, and impaired physical functioning (Chen et al., 2021). The diminished presence of skeletal muscle tissue is a vital element in the identification of sarcopenia, associated with numerous contributors like reduced physical exertion, ongoing inflammation, age-related hormonal alterations, genetic influences, and more (Sk, 2020). Furthermore, Low Skeletal Muscle Mass (LSMM) was considered an autonomous predictor of decreased overall survival in multiple diseases (Walowski et al., 2020). Asia is a rapidly aging region with a large population, and therefore the estimated impact of sarcopenia on this region is substantial. However, due to the lack of standard diagnosis criteria and different target populations used in studies, the prevalence of LSMM varies greatly between studies (Fielding et al., 2011; Chen et al., 2014; Petermann-Rocha et al., 2022). The prevalence of sarcopenia varies between 10% and 27% when using different classifications and cutoff points. According to the European Working Group on Sarcopenia 2 (EWGSOP2) definition, the prevalence of sarcopenia is higher in men (11% vs. 2%). Conversely, as defined by the International Sarcopenia Working Group, the prevalence of sarcopenia is higher in women (17% vs. 12%) (Petermann-Rocha et al., 2022). Analyzing 41 studies and a total of 34,955 participants, another systematic review and meta-analysis showed that among community-dwelling individuals, the prevalence of sarcopenia was 11% in men and 9% in women (Papadopoulou et al., 2020). However, according to several studies, women have physiologically lower levels of skeletal muscle mass than men (Churchward-Venne et al., 2014; Zhang et al., 2021). A cross-sectional study reported that positive serum *H. pylori* infectious markers are correlated with sarcopenia and low muscle quantity (Wu and Chen, 2021). Another study involving 1061 women reported that the risk of low skeletal muscle mass is reduced in older adults women aged 60 and over who receive Helicobacter pylori eradication therapy (Baeg et al., 2015). Skeletal muscle mass begins to decline as early as age 40 and decreases by approximately 30%–50% between ages 40 and 80 (Faulkner et al., 2007). What’s more, LSMM was sex-specific, especially women LSMM appears to be strongly associated with menopause (Yang et al., 2019; Geraci et al., 2021). Therefore, further exploration of the relationship between menopause and low skeletal muscle mass holds significant meaning. In spite of the existing knowledge, comprehensive investigations probing the connection between *H. pylori* and low skeletal muscle mass (LSMM) are scarce. In light of this, this investigation is centered around the female demographic. And the purpose of our study is to determine whether there is a correlation between *H. pylori* infection and LSMM, specifically within a demographic of adult Chinese women. This information could potentially shed light on the interactions between these factors and contribute to a broader understanding of the disease progression and make it possible to take proactive steps to intervene early in order to halt or slow the progression of the disease.

## Methods

### Setting and study population

This research, characterized as a retrospective cohort study, received formal approval from the Ethics Committee associated with the First Affiliated Hospital of Wenzhou Medical University. Furthermore, all research activities and methodologies strictly adhere to the principles outlined in the Declaration of Helsinki. The study encompassed 1984 adult women aged 18 and above who satisfied certain eligibility conditions. To qualify for inclusion, the participants had to be female adults who had undergone a health check at the First Affiliated Hospital of Wenzhou Medical University from April 2016 through August 2017. Additionally, these individuals had to have completed a 13C-urea breath test and bioelectrical impedance analysis (BIA). Our research used the following exclusion criteria: (1) Those under the age of 18 were excluded from the study. (2) had a history of gastrectomy, heart failure, liver cirrhosis, cerebrovascular accident, thyroid disease, end-stage renal disease, or malignancy. (3) physical disability or injuries of hand/wrist/leg/foot in the last three months. (4) using antibiotics and traditional Chinese medicines with antibacterial effects within a month, or proton pump inhibitors (PPI), sucralfate, bismuth, etc within 2 weeks. (5) participants with deficiencies of information such as skeletal muscle mass index, *H. pylori* results, and serum biomarkers.

### Data collection

We collected participants’ basic information, such as age, smoking and drinking status, comorbidities, and past medical history, using pre-designed questionnaires. Smoking was classified as either a current or non-current smoker (someone who has never smoked or used to smoke). Alcohol use was categorized as either a heavy drinker (someone who drinks alcohol at 1 time ≥40 g or 5 drinks, twice or more per week) or not a heavy drinker (someone who drinks less than twice per week). Blood measurements were collected after fasting for 12-16 hours and avoiding alcohol, high-protein, and high-fat foods the day before.

Several blood-based metrics were meticulously evaluated, including counts of white and red blood cells, hemoglobin levels, glucose concentration, glycosylated hemoglobin content, and levels of total protein and albumin. Additionally, measurements of enzymes, Examples of these enzymes include, but are not limited to, alanine aminotransferase and aspartate aminotransferase, in addition to alkaline phosphatase, along with γ-glutamyl transferase, were performed, as were assessments of creatinine, uric acid, total cholesterol, triglycerides, high-density lipoprotein cholesterol, and low-density lipoprotein cholesterol levels. Furthermore, anthropometric parameters were scrupulously recorded by well-trained nursing professionals utilizing uniform and standardized equipment to ensure accuracy and reliability. Blood pressure measurements, presented in millimeters of mercury (mmHg), were conducted in the morning under tranquil and relaxed conditions to minimize external influences. Body weight and height were determined, with weights reported in kilograms and heights noted in centimeters. The formula for calculating BMI (Body Mass Index) is: weight (in kilograms) divided by height squared (in meters).

### Diagnosis of *H. pylori* infection

The 13C-urea breath test (UBT) stands out as a leading non-invasive approach for the identification of *H. pylori* infection. It has straightforward procedural steps and high sensitivity and specificity. The combination of these factors underscores why the 13C-UBT is often the method of choice in clinical settings for the diagnosis of *H. pylori* infection (Nocon et al., 2009; Gisbert and Calvet, 2013; Malfertheiner et al., 2017). After oral administration of nuclide 13C-labeled urea, the urease present in the living *Helicobacter pylori* can break down the nuclide-labeled urea into nuclide-labeled CO_2_. It is necessary to collect and detect the exhaled breath at two time points before taking the medicine and 30 minutes after taking the medicine (Chen et al., 2003; Ricci et al., 2007; Di Rienzo et al., 2013). *H. pylori* infection can be judged by the change in the concentration ratio of ^13^CO_2_/^12^CO_2_ in the breath sample before and after taking the medicine. Generally, Delta Over Baseline (DOB)=4 is used as the cut-off value for *H. pylori* infection, DOB value ≥ 4 means *H. pylori* positive, DOB value < 4 means *H. pylori* negative (Logan, 1998; Gisbert and Pajares, 2004). In this study, *H. pylori* infection status was detected by a 13C-UBT, with an empty stomach or fasting for at least two hours. Participants were required to stop using all kinds of antibiotics and traditional Chinese medicines with antibacterial effects for at least 4 weeks, proton pump inhibitors (PPI), bismuth, sucralfate, etc. for 2 weeks before test.

### Measures and definition of LSMM

Bioelectrical impedance analysis (BIA), an approach doesn’t require invasive procedures that assesses body composition, is recognized by international guidelines as a valid alternative to whole-body dual-energy DXA. In our study, we employed a BIA device (InBody770, produced by InBody Korean Inc.) This method allows for the assessment of appendicular skeletal muscle mass (ASM). Once the ASM was established, we further computed the skeletal muscle index (SMI). This evaluation was performed by taking the previously acquired value for appendicular skeletal muscle mass and dividing it by the participant’s height squared, with the height being measured in square meters (m^2^). To define LSMM, we adhered to the unified recommendations put forth by the Asian Working Group for Sarcopenia (AWGS) in 2019. According to these guidelines, the thresholds of LSMM were an SMI below 7.0 kg/m^2^ for men and below 5.7 kg/m^2^ for women. These cutoff values are widely accepted and used in sarcopenia research, enabling the identification of individuals at risk or already affected by the condition (Chen et al., 2020).

### Statistical analyses

All our statistical computations were performed employing a two-tailed approach and were conducted with a 5% significance level, utilizing SPSS statistical software (version 23.0 provided by IBM Corp). The normality of the continuous variables within our dataset was validated using the Kolmogorov-Smirnov test. For those variables adhering to a normal distribution, they were presented as means ± standard deviations. To discern any disparities between the two groups, we implemented the Student’s t-test. For the continuous variables that didn’t follow a normal distribution, we presented them as medians, alongside their interquartile ranges. To compare differences between these two groups, we utilized the Mann-Whitney U test. When it came to categorical variables, we expressed them as frequencies and percentages. To identify the differences between the two groups in this context, we applied the chi-square test or, when necessary, the exact method proposed by Fisher. For evaluating the ORs and their associated 95% CIs across various groups, both univariate and multivariate-adjusted calculations were made, deploying logistic regression analysis.

## Results

### Characteristics of participants

**Table 1** provides a snapshot of the primary characteristics of our study subjects, divided based on their *H. pylori* infection condition as identified via the 13C-urea breath test. The study included a total of 1984 fitting participants, with their average age being 46.67 ± 10.70 years. Among the study population, the mean ages of the Helicobacter pylori-negative and Helicobacter pylori-positive group were 47.03 ± 10.89, 46.29 ± 10.48. No significant age differences were observed between the two groups under study (t=1.529, *p*=0.127). The total incidence of *H. pylori* infection was found to be 48.0% (953 out of 1984 participants). The distribution of this infection did not present notable statistical disparities when comparing the group with LSMM and the group without LSMM (15.2% vs 17.1%, *p*=0.256). Thus, the prevalence of *H. pylori* infection does not seem to be biased towards one group over the other, indicating a balanced sample for our investigation. Most parameters, such as smoking status, alcohol status, age, BMI, SBP, DBP, RBC, Hb, TP, GLU, ALT, AST, ALK, UA, TC, TG, HDL-C, LDL-C showed no notable statistical disparities between individuals with *H. pylori* infection and those without. Nonetheless, variables such as WBC (P <0.001), Ghb (*p*= 0.043), ALB (*p*= 0.003), Cre (*p*= 0.040), did present statistical differences (*p*<0.05).

**Table 1:** Initial attributes of the study participants based on their *H. pylori* infection condition.

Characteristics		Total (n = 1984,100%)	*H. pylori* infection (-) (n=1031,52.0%)	*H. pylori* infection (+) (n= 953,48.0%)	*p-value*
LSMM	No	1664 (83.9%)	874 (84.8%)	790 (82.9%)	0.256
	Yes	320 (16.1%)	157 (15.2%)	163 (17.1%)	
Smoke	No	1961 (98.8%)	1021 (99.0%)	940 (98.6%)	0.413
	Yes	23 (1.2%)	10 (1.0%)	13 (1.4%)	
Drink	No	1777 (89.6%)	933 (90.5%)	844 (88.6%)	0.160
	Yes	207 (10.4%)	98 (9.5%)	109 (11.4%)	
Age (years)		46.67 ± 10.70	47.03 ± 10.89	46.29 ± 10.48	0.127
BMI (kg/m^2^)		22.56 ± 2.96	22.50 ± 2.92	22.63 ± 3.01	0.347
SBP (mmHg)		119.92 ± 19.49	120.29 ± 19.19	119.52 ± 19.80	0.377
DBP (mmHg)		68.24 ± 11.93	68.37 ± 11.47	68.11 ± 12.41	0.631
WBC (10^9/L)		5.64 ± 1.53	5.48 ± 1.39	5.81 ± 1.65	**<0.001**
RBC (10^12/L)		4.46 ± 0.35	4.46 ± 0.34	4.45 ± 0.36	0.313
Hb (g/L)		131.20 ± 29.87	131.05 ± 11.85	131.35 ± 41.30	0.823
GLU (mmol/L)		4.88 ± 1.18	4.86 ± 1.12	4.90 ± 1.24	0.467
Ghb (%)		5.50 ± 0.76	5.47 ± 0.68	5.54 ± 0.83	**0.043**
TP (g/L)		74.43 ± 4.61	74.57 ± 4.64	74.28 ± 4.57	0.159
ALB (g/L)		44.09 ± 2.92	44.27 ± 2.91	43.89 ± 2.93	**0.003**
ALT (U/L)		16.00 (13.00,22.00)	16.00 (13.00,22.00)	17.00 (13.00,23.00)	0.247
AST (U/L)		21.00 (18.00,25.00)	21.00 (18.00,25.00)	21.00 (18.00,26.00)	0.743
ALK (U/L)		65.00 (53.00,81.00)	65.00 (53.00,81.00)	64.00 (51.00,81.00)	0.262
GGT (U/L)		17.00 (14.00,25.00)	17.00 (13.00,24.00)	17.00 (14.00,26.00)	0.073
Cre (umol/L)		55.13 ± 8.08	55.49 ± 8.17	54.74 ± 7.97	**0.040**
UA (umol/L)		281.06 ± 62.15	280.33 ± 61.24	281.86 ± 63.15	0.583
TC (mmol/L)		5.16 ± 1.07	5.13 ± 1.03	5.20 ± 1.10	0.119
TG (mmol/L)		1.39 ± 1.00	1.39 ± 0.94	1.39 ± 1.06	0.971
HDL-C (mmol/L)		1.41 ± 0.36	1.41 ± 0.35	1.42 ± 0.37	0.575
LDL-C (mmol/L)		3.04 ± 0.86	3.01 ± 0.81	3.06 ± 0.90	0.209

For clarity and ease of understanding, the following abbreviations have been employed throughout the text: LSMM stands for Low Skeletal Muscle Mass; BMI, an abbreviation for Body Mass Index, or BMI, is determined by dividing a person’s weight, measured in kilograms, by their height in centimeters squared. Meanwhile, SBP stands for Systolic Blood Pressure and DBP signifies Diastolic Blood Pressure. In terms of blood components, WBC and RBC are acronyms representing White Blood Cells and Red Blood Cells, respectively. Hb is short for Hemoglobin; GLU signifies Glucose; Ghb is used for Glycosylated Hemoglobin; TP represents Total Protein; ALB is the shorthand for Albumin; ALT and AST stand for Alanine Aminotransferase and Aspartate Aminotransferase respectively; ALK is the abbreviation for Alkaline Phosphatase; GGT is used for γ-glutamyl Transferase; Cre is the short form for Creatinine; UA denotes Uric Acid; TC is the abbreviation for Total Cholesterol; TG refers to Triglycerides; HDL-C and LDL-C stand for High-Density Lipoprotein Cholesterol and Low-Density Lipoprotein Cholesterol respectively. For your convenience, entries that are bolded indicate a p-value that is less than 0.05, thus signifying statistical significance in our analysis.

### Logistic regression analyses for different markers and LSMM

The univariate logistic regression analysis results revealed notable correlations between LSMM and various factors. These include Alcohol Consumption (OR=0.456, 95% CI 0.282-0.766, *p*=0.003), BMI (OR=0.566, 95% CI 0.527-0.607, *p*<0.001), SBP (OR=0.991, 95% CI 0.984-0.997, *p*=0.005), WBC (OR=0.919, 95% CI 0.847-0.998, *p*=0.044), RBC (OR=0.649, 95% CI 0.458-0.918, *p*=0.015), Glu (OR=0.770, 95% CI 0.659-0.900, *p*<0.001), Ghb (OR=0.749 95% CI 0.608-0.926, *p*=0.007), ALB (OR=1.038, 95% CI 0.996-1,082, *p*=0.079), ALK (OR=1.004, 95% CI 1.001-1,008, *p*=0.011), Cre (OR=0.975, 95% CI 0.960-0.990, *p*=0.001), UA (OR=0.995, 95% CI 0.993-0.998, *p*<0.001), TG (OR=0.805, 95% CI 0.683-0.949, *p*=0.010), and HDL-C (OR=1.743, 95% CI 1.273-2.387, *p*=0.001).

**Figure 1:**
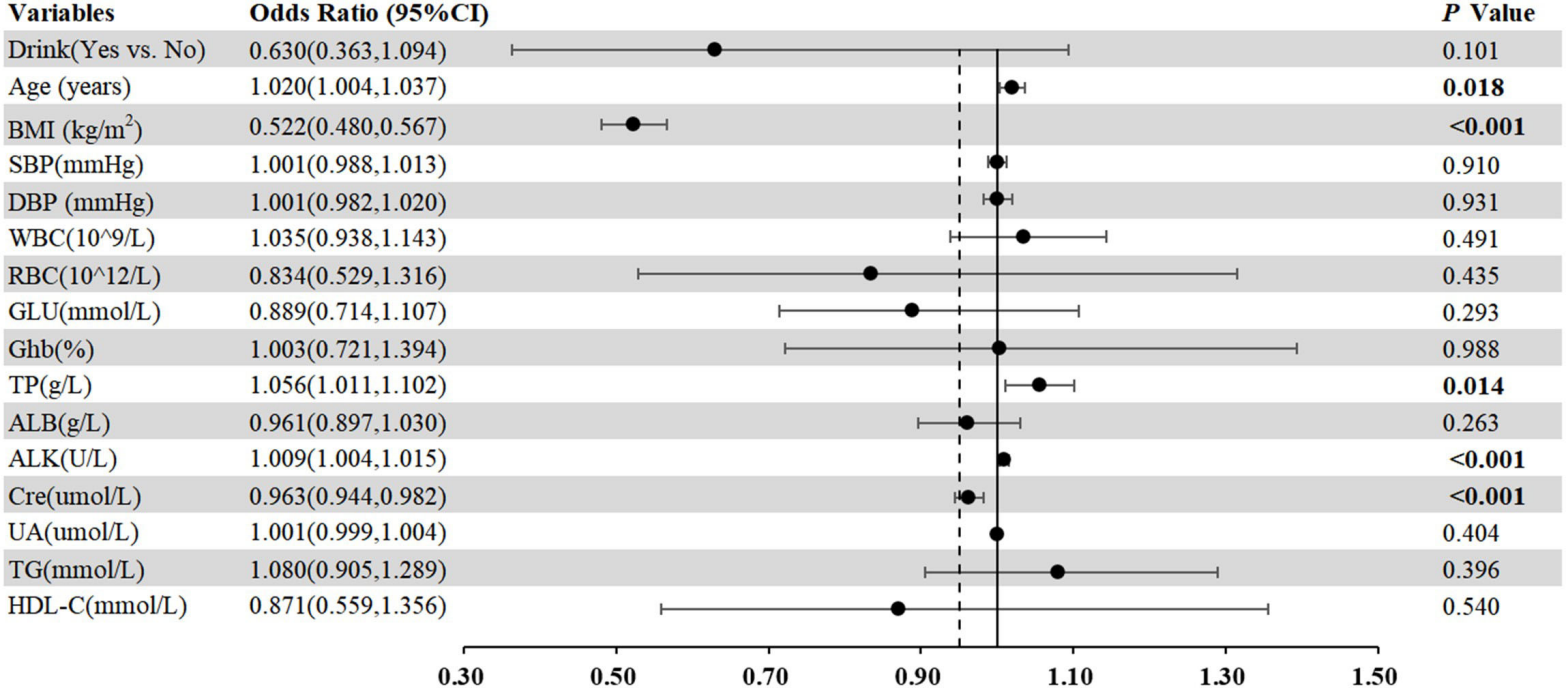
Forest plots of multivariate analysis with 95% CI representing pooled estimates for the association between different markers and LSMM.

As displayed in **Table 2**, no significant relationship was found between *H. pylori* infection status and LSMM, corroborated by univariate analyses (*p*=0.257). Additionally, we conducted multivariate analyses including variables that were clinically relevant and had a *P* value < 0.1 in univariate analysis, such as Drink, BMI, SBP, WBC, RBC, Glu, Ghb, ALB, ALK, Cre, UA, TG and HDL-C. Our results indicated a significant association between LSMM and age (OR=1.020, 95% CI 1.004-1.037, *p*<0.001), BMI (OR=0.522, 95% CI 0.480-0.576, *p<*0.001), TP (OR=1.056, 95% CI 1.011-1.012, *p=*0.014), ALK (OR=1.009, 95% CI 1.004-1.015, *p<*0.001) and Cre (OR=0.965, 95% CI 0.947-0.984, *p<*0.001). To enhance the understanding of these associations, we have also visualized these findings in a forest plot (**Figure 1**).

**Table 2:** Logistic regression analyses of various markers in relation to LSMM.

Independent variables	Crude OR (95% CI)	*p-value*	Adjusted OR (95% CI)	*p-value*
*H. pylori* infection (No vs. Yes)	1.149 (0.904,1.459)	0.257		
Smoke (Yes vs. No)	0.492 (0.115,2.109)	0.340		
Drink (Yes vs. No)	0.465 (0.282,0.766)	**0.003**	0.630 (0.363,1.094)	0.101
Age (years)	0.990 (0.979,1.002)	0.093	1.020 (1.004,1.037)	**0.018**
BMI (kg/m^2^)	0.566 (0.527,0.607)	**<0.001**	0.522 (0.480,0.567)	**<0.001**
SBP (mmHg)	0.991 (0.984,0.997)	**0.005**	1.001 (0.988,1.013)	0.910
DBP (mmHg)	0.991 (0.981,1.001)	0.071	1.001 (0.982,1.020)	0.931
WBC (10^9/L)	0.919 (0.847,0.998)	**0.044**	1.035 (0.938,1.143)	0.491
RBC (10^12/L)	0.649 (0.458,0.918)	**0.015**	0.834 (0.529,1.316)	0.435
Hb (g/L)	0.998 (0.990,1.005)	0.586		
GLU (mmol/L)	0.770 (0.659,0.900)	**0.001**	0.889 (0.714,1.107)	0.293
Ghb (%)	0.749 (0.608,0.926)	**0.007**	1.003 (0.721,1.394)	0.988
TP (g/L)	1.025 (0.999,1.052)	0.059	1.056 (1.011,1.102)	**0.014**
ALB (g/L)	1.038 (0.996,1.082)	**0.079**	0.961 (0.897,1.030)	0.263
ALT (U/L)	1.000 (0.998,1.003)	0.751		
AST (U/L)	1.001 (0.996,1.006)	0.628		
ALK (U/L)	1.004 (1.001,1.008)	**0.011**	1.009 (1.004,1.015)	**<0.001**
GGT (U/L)	0.998 (0.992,1.003)	0.385		
Cre (umol/L)	0.975 (0.960,0.990)	**0.001**	0.963 (0.944,0.982)	**<0.001**
UA (umol/L)	0.995 (0.993,0.998)	**<0.001**	1.001 (0.999,1.004)	0.404
TC (mmol/L)	1.049 (0.939,1.172)	0.400		
TG (mmol/L)	0.805 (0.683,0.949)	**0.010**	1.080 (0.905,1.289)	0.396
HDL-C (mmol/L)	1.743 (1.273,2.387)	**0.001**	0.871(0.559,1.356)	0.540
LDL-C (mmol/L)	0.975 (0.847,1.122)	0.721		

Entries marked in bold typeface denote a p-value under 0.05, which is universally acknowledged as reaching the threshold for statistical significance.

Regarding the odds ratios, they have been adjusted in the context of multivariate analyses. The adjustment includes those variables which, upon univariate analysis, showed a significance level of p < 0.1 and were also deemed to bear clinical relevance, thus warranting their inclusion in the multivariate analyses.

### The relationship between LSMM and *H. pylori* infection in different age group

There was not a significant disparity in the presence of LSMM when comparing the group with *H. pylori* infection and the group without. Nevertheless, when we dissected the data based on age brackets (as depicted in **Table 3**), a compelling link was found between *H. pylori* infection status and LSMM among females aged 40 years or older (OR=1.381, 95%CI 1.032-1.848, *p* = 0.030). This correlation persisted, remaining statistically significant (OR=1.514, 95%CI 1.085-2.113, *p* = 0.015), even after adjustments were made for several variables (Age, BMI, TP, ALK, Cre). More specifically, our analysis indicated that women aged 40 or older with *H. pylori* infection displayed an elevated occurrence of LSMM. Conversely, no statistical divergence was observed in relation to *H. pylori* infection status and LSMM in women younger than 40 years.

**Table 3:** The correlation between LSMM and *H. pylori* infection across diverse age categories.

	*H. pylori* (-)	*H. pylori* (+)	Crude OR (95% CI)	*p-value*	Adjusted OR (95% CI)	*p-value*
age≥40 years old						
LSMM (-)	679 (87.4%)	577 (83.4%)	1.381 (1.032,1.848)	**0.030**	1.514 (1.085,2.113)	**0.015**
LSMM (+)	98 (12.6%)	115 (16.6%)				
Age<40 years old						
LSMM (-)	195 (76.8%)	213 (81.6%)	0.745 (0.486,1.142)	0.177	0.912 (0.543,1.532)	0.729
LSMM (+)	59 (23.2%)	48 (18.4%)				

Bold entries indicate p< 0.05, which is statistically significant.

Adjusted OR (Adjusted for Age, BMI, TP, ALK, Cre).

### The relationship between different age group and markers

We stratified the study population according to age and further analyzed the baseline characteristics, such as metabolic syndrome related indicators (triglycerides, HDL-C, blood pressure, hyperglycaemia). As delineated in **Table 4**, significant variances were observed in several markers such as smoking habits, age, BMI, blood pressure, glucose levels, white blood cell count, hemoglobin, liver function tests (ALT, AST, ALK, and GGT), creatinine levels, uric acid levels, and lipid profiles (TC, TG, HDL-C, and LDL-C) across different age groups.

**Table 4:** Initial Attributes of the Patients Across Various Age Categories.

Characteristics		Total (n = 1984,100%)	age<40 years old (n=515,26.0%)	age≥40 years old (n= 1469,74.0%)	*p*-value
HP infection	No	1031 (52.0%)	254 (49.3%)	777 (52.9%)	0.163
	Yes	953 (48.0%)	261 (50.7%)	692 (47.1%)	
Smoke	No	1961 (98.8%)	503 (97.7%)	1458 (99.3%)	**0.004**
	Yes	23 (1.2%)	12 (2.3%)	11 (0.7%)	
Drink	No	1777 (89.6%)	453 (88.0%)	1324 (90.1%)	0.166
	Yes	207 (10.4%)	62 (12.0%)	145 (9.9%)	
Age (years)		46.67 ± 10.70	33.98 ± 4.32	51.12 ± 8.47	<**0.001**
BMI (kg/m^2^)		22.56 ± 2.96	21.36 ± 2.78	22.99 ± 2.91	<**0.001**
SBP (mmHg)		119.92 ± 19.49	109.83 ± 13.85	123.46 ± 19.94	<**0.001**
DBP (mmHg)		68.24 ± 11.93	64.55 ± 10.61	69.54 ± 12.10	<**0.001**
WBC (10^9/L)		5.64 ± 1.53	5.77 ± 1.57	5.60 ± 1.51	**0.026**
RBC (10^12/L)		4.46 ± 0.35	4.48 ± 0.33	4.45 ± 0.36	0.095
Hb (g/L)		131.20 ± 29.87	133.49 ± 54.31	130.39 ± 13.02	**0.043**
GLU (mmol/L)		4.88 ± 1.18	4.65 ± 0.99	4.96 ± 1.23	<**0.001**
Ghb (%)		5.50 ± 0.76	5.24 ± 0.64	5.60 ± 0.77	<**0.001**
TP (g/L)		74.43 ± 4.61	74.26 ± 4.82	74.49 ± 4.53	0.325
ALB (g/L)		44.09 ± 2.92	44.49 ± 3.06	43.95 ± 2.86	<**0.001**
ALT (U/L)		16.00 (13.00,22.00)	14.00 (11.00,20.00)	17.00 (13.00,23.00)	<**0.001**
AST (U/L)		21.00 (18.00,25.00)	19.00 (17.00,23.00)	22.00 (19.00,26.00)	<**0.001**
ALK (U/L)		65.00 (53.00,81.00)	57.00 (48.00,66.00)	68.00 (55.00,85.50)	<**0.001**
GGT (U/L)		17.00 (14.00,25.00)	15.00 (12.00,19.00)	18.00 (14.00,27.00)	<**0.001**
Cre (umol/L)		55.13 ± 8.08	53.87 ± 6.76	55.57 ± 8.45	<**0.001**
UA (umol/L)		281.06 ± 62.15	271.01 ± 55.75	284.59 ± 63.89	<**0.001**
TC (mmol/L)		5.16 ± 1.07	4.72 ± 0.93	5.32 ± 1.07	<**0.001**
TG (mmol/L)		1.39 ± 1.00	1.11 ± 0.99	1.48 ± 0.98	<**0.001**
HDL-C (mmol/L)		1.41 ± 0.36	1.47 ± 0.35	1.39 ± 0.36	<**0.001**
LDL-C (mmol/L)		3.04 ± 0.86	2.66 ± 0.71	3.17 ± 0.86	<**0.001**

Bold entries indicate p< 0.05, which is statistically significant.

## Discussion

In summary, our study has shed light on several critical risk factors associated with LSMM. Through rigorous univariate and multivariate logistic regression analysis, we had identified age, BMI, TP, ALK and Cre as significant risk factors that are closely linked to this disease. These results represent significant additions and provide further insights into the study of risk factors in LSMM. Nonetheless, the crux of this study reveals a meaningful association between *H. pylori* infection and LSMM in Chinese women aged over 40 years. This implies that women within this demographic who are infected with *H. pylori* could have an elevated likelihood of LSMM development. The potential mechanisms explaining the harmful effects of *H. pylori* infection on LSMM could be as follows. Numerous studies had suggested that inflammation response might play vital role in loss of skeletal muscle (Ryall et al., 2008; Londhe and Guttridge, 2015; Dhillon and Hasni, 2017; Howard et al., 2020). For instance, research examining the relationship between older adults sarcopenia and inflammatory factors revealed that patients with sarcopenia had significantly increased circulating concentrations of IL-6 and TNFα (Bian et al., 2017). Additional research has suggested that TNF-α and IL-6 play a significant role in the inflammatory response of skeletal muscle, with elevated levels of IL-6 being associated with reductions in both muscle mass and strength (Wang et al., 2017). The release of the Vacuolating cytotoxin A (VacA) protein plays a crucial role in *H. pylori* infection, as it initiates the generation of multiple proinflammatory cytokines. These include macrophage-inflammatory protein-1α, TNF-α, along with interleukins such as IL-1β, IL-6, IL-10, and IL-13 (Watanabe et al., 2010; Figueiredo et al., 2014). Additionally, the impact of early environment on growth and development may have long-term effects on humans. A prospective cohort study strongly pointed out that *H. pylori* infection may hinder linear growth in preschool children (Bravo et al., 2003), leading to reduced absorption of important nutrients and slow growth in children, ultimately resulting in low skeletal muscle mass in older adults individuals (Franceschi et al., 2014). What’s more, ghrelin and leptin may also be factors contributing to the association between *H. pylori* and LSMM, as *H. pylori* has been shown to affect their secretion (Meng et al., 2016). Leptin, a hormone secreted by adipose tissue, performs a pivotal function in various physiological processes. These include inflammation regulation, insulin sensitivity, appetite regulation, and the deposition of fat (Lin et al., 2018), while ghrelin can increase muscle mass through stimulating appetite, increasing food intake, promoting myocyte differentiation and fusion, among other ways (Baeg et al., 2015). And several studies showed decreased levels of ghrelin and leptin in patients with LSMM (Kohara et al., 2011; Serra-Prat et al., 2015).

However, there was no statistically significant variation observed among women of all ages. Further analysis conducted on subgroups revealed that LSMM was only linked to *H. pylori* infection in women aged 40 years and above. Maybe sex hormones were responsible for age-specific differences. Some researches had demonstrated the protective effect of certain steroids against *H. pylori* infection (Hosoda et al., 2011; Ohtani et al., 2011; Fong and Wang, 2021). For example, Kouichi Hosoda discovered that the growth of *H. pylori* could be inhibited by estradiol and progesterone. Among the two hormones, progesterone exhibited the strongest antimicrobial effect (Hosoda et al., 2011). In a study conducted by P Fong, the protective effect of oral contraceptives against *H. pylori* infection was investigated. The results indicated that women who were using oral contraceptives had a significantly reduced risk of acquiring *H. pylori* infection in comparison to those who did not use them (Fong and Wang, 2021). As women reach the age of 40, their levels of free estradiol decline significantly, which weakens the protective effect of hormones on them (Runolfsdottir et al., 2015).This may lead to a more vulnerable state in older women, resulting in greater muscle decline following *H. pylori* infection. Moreover, after analyzing the study population, we found that there were significant differences in metabolic syndrome related indicators, including BMI, blood pressure, TC, TG between different age groups. This may also explain why a significant link between *H. pylori* infection and LSMM was only observed in women aged 40 or older. Metabolic syndrome is also called syndrome X or insulin resistance syndrome, which is a cluster of metabolic abnormalities including abdominal obesity, dysglycemia, elevated blood pressure, low HDL cholesterol and high triglycerides (Saklayen, 2018; Lemieux and Després, 2020). Metabolic syndrome is a group of conditions that raise risk of serious health problems such as coronary heart disease, type 2 diabetes, stroke, and even all-cause mortality (Isomaa et al., 2001; Grundy, 2008). Many studies have linked sarcopenia to various metabolic disorders, such as high blood pressure, dyslipidemia, dysglycemia (Doğan et al., 2012; Baek et al., 2014; Nishikawa et al., 2021). Several previous studies have revealed a relationship between metabolic syndrome and sarcopenia. Metabolic syndrome may be associated with muscle loss through a complex interplay of multiple factors, including pro-inflammatory cytokines, insulin resistance, mitochondrial dysfunction and oxidative stress (Sanada et al., 2012; Moon, 2014; Rubio-Ruiz et al., 2019; Gonzalez et al., 2021).

Our results underscore the significance of incorporating these elements when investigating the link between *H. pylori* and LSMM across various age brackets. Future studies can utilize these findings as a foundation to delve deeper into the age-specific disparities associated with the influence of *H. pylori* infection on muscle deterioration, and to pinpoint potential preventive measures or treatments to alleviate its consequences.

Up until now, there has been scant investigation into the correlation between *H. pylori* infection and LSMM. That being said, in a study conducted with a sample size of 3453 American individuals aged 60 and over indicated an association between positive markers of *H. pylori* infection in serum and the presence of sarcopenia along with reduced muscle mass (Wu and Chen, 2021). Although this study had a larger number of participants, it only included American people aged ≥60 years. Our study included Asian populations, which are different from the ethnic groups they studied. What’s more, based on the available evidence, it is estimated that more than 1 in every 10 young adults from various ethnicities may have sarcopenia (Jung et al., 2023). Thus, it is imperative to associate sarcopenia not only with frail older patients but also with younger patients. So, we selected the physical examination population aged >18 years as the inclusion population.

Another study compared healthy, asymptomatic women who had received *H. pylori* eradication treatment with those who were HP IgG positive but had not undergone eradication therapy. This study found that older adults women who underwent *H. pylori* eradication had a reduced risk of LSMM (Baeg et al., 2015). It is worth mentioning that both of these studies utilized serological testing as the method for determining the status of *H. pylori* infection. While this method is commonly used, it requires validation for specific locations and may produce false results due to cross-reactivity. Additionally, serology antibody levels often persist for several years after therapy, which can make it difficult to accurately assess current infection status and disease outcomes (Katelaris et al., 2023). In contrast, our study utilized the 13C-urea breath test to diagnose active *H. pylori* infection, which is considered a reliable technique for detecting active infections (Huh and Kim, 2018). It is widely recognized that urea breath tests (UBTs) are more accurate than other non-invasive tests in patients who have not undergone gastrectomy (Katelaris et al., 2023).Furthermore, it is important to acknowledge that our research has some limitations. Firstly, due to its retrospective nature, there may have been some selection bias in the study population. Additionally, we did not collect data on certain critical factors related to LSMM, such as muscle strength and gait speed. And we also did not collect some important data such as sex hormone levels. Hence, future research endeavors should strive to incorporate a more extensive sample size and meticulous data collection in order to delve deeper into the association between *H. pylori* infection and LSMM. By doing so, we can gain a more complete understanding of this complex issue and develop better strategies for managing and preventing LSMM in particular individuals.

## Conclusions

Our study did not conclusively show a connection between *H. pylori* infection and LSMM among all women. Interestingly, our findings indicated a potential link between *H. pylori* infection and LSMM in women aged over 40. It suggests that there is a certain correlation between menopause and low skeletal muscle mass in women. And this underscores the significance of early detection and remediation of *H. pylori* infection as a possible tactic for warding off LSMM in this demographic. A more profound exploration is required to decipher the mechanisms behind this association and how it fluctuates across distinct populations. The importance of pursuing this research topic lies in forming more potent approaches to managing and mitigating LSMM, especially in high-risk individuals due to factors like age or sex. In summary, while our study didn’t present definitive proof, it hinted at a possible association between *H. pylori* infection and LSMM in certain demographics. Future research should strive to delve deeper into this relationship, factoring in elements such as dietary habits, physical activity level and sex hormone levels, to achieve a holistic understanding of this concern.

## Data availability statement

The raw data supporting the conclusions of this article will be made available by the authors, without undue reservation.

## Ethics statement

The studies involving humans were approved by The Ethics Committee of the First Affiliated Hospital of Wenzhou Medical University. The studies were conducted in accordance with the local legislation and institutional requirements. Written informed consent for participation was not required from the participants or the participants’ legal guardians/next of kin in accordance with the national legislation and institutional requirements. Written informed consent was obtained from the individual(s) for the publication of any potentially identifiable images or data included in this article.

## Author contributions

XX: Writing – original draft, Formal analysis, Conceptualization, Methodology. YQ: Formal analysis, Writing – original draft, Conceptualization, Investigation. KJ: Data curation, Formal analysis, Resources, Writing – original draft. JC: Resources, Validation, Writing – original draft. JF: Data curation, Writing – original draft. CC: Conceptualization, Writing – review & editing, Funding acquisition, Supervision. ZZ: Supervision, Writing – review & editing, Conceptualization, Project administration.

## References

[B1] Baeg M. K. Choi M.-G. Ko S.-H. Lim C.-H. Kim J. S. Cho Y. K.. (2015). Elderly women who received Helicobacter pylori-eradicating therapy have reduced risk of low skeletal muscle mass. Clin. Interv Aging 10, 1771–1777. doi: 10.2147/CIA.S95007 26586939 PMC4634827

[B2] Baek S. J. Nam G. E. Han K. D. Choi S. W. Jung S. W. Bok A. R.. (2014). Sarcopenia and sarcopenic obesity and their association with dyslipidemia in Korean elderly men: the 2008-2010 Korea National Health and Nutrition Examination Survey. J. Endocrinol. Invest. 37, 247–260. doi: 10.1007/s40618-013-0011-3 24615361

[B3] Bian A.-L. Hu H.-Y. Rong Y.-D. Wang J. Wang J.-X. Zhou X.-Z. (2017). A study on relationship between elderly sarcopenia and inflammatory factors IL-6 and TNF-α. Eur. J. Med. Res. 22, 25. doi: 10.1186/s40001-017-0266-9 28701179 PMC5508730

[B4] Bravo D. Hoare A. Soto C. Valenzuela M. A. Quest A. F. (2018). Helicobacter pylori in human health and disease: Mechanisms for local gastric and systemic effects. World J. Gastroenterol. 24, 3071–3089. doi: 10.3748/wjg.v24.i28.3071 30065554 PMC6064966

[B5] Bravo L. E. Mera R. Reina J. C. Pradilla A. Alzate A. Fontham E.. (2003). Impact of Helicobacter pylori infection on growth of children: a prospective cohort study. J. Pediatr. Gastroenterol. Nutr. 37, 614–619. doi: 10.1097/00005176-200311000-00021 14581807

[B6] Buzás G. M. (2020). Helicobacter pylori and non-alcoholic fatty liver disease. Minerva Gastroenterol. Dietol 66, 267–279. doi: 10.23736/S1121-421X.20.02671-9 32724031

[B7] Chen T.-P. Hung H.-F. Chen M.-K. Lai H.-H. Hsu W.-F. Huang K.-C.. (2015). Helicobacter pylori infection is positively associated with metabolic syndrome in Taiwanese adults: a cross-sectional study. Helicobacter 20, 184–191. doi: 10.1111/hel.12190 25582223

[B8] Chen T.-S. Chang F.-Y. Chen P.-C. Huang T. W. Ou J. T. Tsai M.-H.. (2003). Simplified 13C-urea breath test with a new infrared spectrometer for diagnosis of Helicobacter pylori infection. J. Gastroenterol. Hepatol. 18, 1237–1243. doi: 10.1046/j.1440-1746.2003.03139.x 14535979

[B9] Chen Z. Li W.-Y. Ho M. Chau P.-H. (2021). The prevalence of sarcopenia in Chinese older adults: meta-analysis and meta-regression. Nutrients 13, 1441. doi: 10.3390/nu13051441 33923252 PMC8146971

[B10] Chen L. K. Liu L. K. Woo J. Assantachai P. Auyeung T.-W. Bahyah K. S.. (2014). Sarcopenia in Asia: consensus report of the Asian Working Group for Sarcopenia. J. Am. Med. Directors Assoc. 15, 95–101. doi: 10.1016/j.jamda.2013.11.025 24461239

[B11] Chen L.-K. Woo J. Assantachai P. Auyeung T.-W. Chou M.-Y. Iijima K.. (2020). Asian working group for sarcopenia: 2019 consensus update on sarcopenia diagnosis and treatment. J. Am. Med. Dir Assoc. 21, 300–307.e2. doi: 10.1016/j.jamda.2019.12.012 32033882

[B12] Churchward-Venne T. A. Breen L. Phillips S. M. (2014). Alterations in human muscle protein metabolism with aging: Protein and exercise as countermeasures to offset sarcopenia. Biofactors 40, 199–205. doi: 10.1002/biof.1138 24105883

[B13] Cover T. L. Blaser M. J. (2009). Helicobacter pylori in health and disease. Gastroenterology 136, 1863–1873. doi: 10.1053/j.gastro.2009.01.073 19457415 PMC3644425

[B14] Dhillon R. J. S. Hasni S. (2017). Pathogenesis and management of sarcopenia. Clin. Geriatr. Med. 33, 17–26. doi: 10.1016/j.cger.2016.08.002 27886695 PMC5127276

[B15] Di Rienzo T. A. D’Angelo G. Ojetti V. Campanale M. C. Tortora A. Cesario V.. (2013). 13C-Urea breath test for the diagnosis of Helicobacter pylori infection. Eur. Rev. Med. Pharmacol. Sci. 17 (Suppl 2), 51–58. 24443069

[B16] Doğan M. H. Karadag B. Ozyigit T. Kayaoglu S. Ozturk A. O. Altuntas Y. (2012). Correlations between sarcopenia and hypertensive target organ damage in a Turkish cohort. Acta Clin. Belg 67, 328–332. doi: 10.2143/ACB.67.5.2062685 23189539

[B17] Faulkner J. A. Larkin L. M. Claflin D. R. Brooks S. V. (2007). Age-related changes in the structure and function of skeletal muscles. Clin. Exp. Pharmacol. Physiol. 34, 1091–1096. doi: 10.1111/j.1440-1681.2007.04752.x 17880359

[B18] Fielding R. A. Vellas B. Evans W. J. Bhasin S. Morley J. E. Newman A. B.. (2011). Sarcopenia: an undiagnosed condition in older adults. Current consensus definition: prevalence, etiology, and consequences. International working group on sarcopenia. J. Am. Med. Dir Assoc. 12, 249–256. doi: 10.1016/j.jamda.2011.01.003 21527165 PMC3377163

[B19] Figueiredo C. A. Marques C. R. Costa R dos S. da Silva H. B. F. Alcantara-Neves N. M. (2014). Cytokines, cytokine gene polymorphisms and Helicobacter pylori infection: friend or foe? World J. Gastroenterol. 20, 5235–5243. doi: 10.3748/wjg.v20.i18.5235 24833853 PMC4017038

[B20] Fong P. Wang Q. T. (2021). Protective effect of oral contraceptive against Helicobacter pylori infection in US adult females: NHANES 1999-2000. Epidemiol. Infect. 149, e120. doi: 10.1017/S0950268821000923 33896437 PMC8161376

[B21] Franceschi F. Annalisa T. Teresa D. R. Giovanna D. Ianiro G. Franco S.. (2014). Role of Helicobacter pylori infection on nutrition and metabolism. World J. Gastroenterol. 20, 12809–12817. doi: 10.3748/wjg.v20.i36.12809 25278679 PMC4177464

[B22] Franceschi F. Gasbarrini A. (2007). Helicobacter pylori and extragastric diseases. Best Pract. Res. Clin. Gastroenterol. 21, 325–334. doi: 10.1016/j.bpg.2006.10.003 17382280

[B23] Geraci A. Calvani R. Ferri E. Marzetti E. Arosio B. Cesari M. (2021). Sarcopenia and menopause: the role of estradiol. Front. Endocrinol. (Lausanne) 12. doi: 10.3389/fendo.2021.682012 PMC8170301 34093446

[B24] Gisbert J. P. Calvet X. (2013). Helicobacter pylori “Test-and-treat” Strategy for management of dyspepsia: A comprehensive review. Clin. Transl. Gastroenterol. 4, e32. doi: 10.1038/ctg.2013.3 23535826 PMC3616453

[B25] Gisbert J. P. Pajares J. M. (2004). Review article: 13C-urea breath test in the diagnosis of Helicobacter pylori infection – a critical review. Aliment Pharmacol. Ther. 20, 1001–1017. doi: 10.1111/j.1365-2036.2004.02203.x 15569102

[B26] Gonzalez A. Simon F. Achiardi O. Vilos C. Cabrera D. Cabello-Verrugio C. (2021). The critical role of oxidative stress in sarcopenic obesity. Oxid. Med. Cell Longev 2021, 4493817. doi: 10.1155/2021/4493817 34676021 PMC8526202

[B27] Grundy S. M. (2008). Metabolic syndrome pandemic. Arterioscler. Thromb. Vasc. Biol. 28, 629–636. doi: 10.1161/ATVBAHA.107.151092 18174459

[B28] Hosoda K. Shimomura H. Hayashi S. Yokota K. Hirai Y. (2011). Steroid hormones as bactericidal agents to Helicobacter pylori. FEMS Microbiol. Lett. 318, 68–75. doi: 10.1111/j.1574-6968.2011.02239.x 21306429

[B29] Howard E. E. Pasiakos S. M. Blesso C. N. Fussell M. A. Rodriguez N. R. (2020). Divergent roles of inflammation in skeletal muscle recovery from injury. Front. Physiol. 11. doi: 10.3389/fphys.2020.00087 PMC7031348 32116792

[B30] Huh C. W. Kim B.-W. (2018). [Diagnosis of helicobacter pylori infection]. Korean J. Gastroenterol. 72, 229–236. doi: 10.4166/kjg.2018.72.5.229 30642138

[B31] Hunt R. H. Xiao S. D. Megraud F. Leon-Barua R. Bazzoli F. van der Merwe S.. (2011). World gastroenterology organisation global guideline: Helicobacter pylori in developing countries. J. Dig. Dis. 12, 319–326. doi: 10.1111/j.1751-2980.2011.00529.x 21955424

[B32] Isomaa B. Almgren P. Tuomi T. Forsén B. Lahti K. Nissén M.. (2001). Cardiovascular morbidity and mortality associated with the metabolic syndrome. Diabetes Care 24, 683–689. doi: 10.2337/diacare.24.4.683 11315831

[B33] Jung H. N. Jung C. H. Hwang Y.-C. (2023). Sarcopenia in youth. Metabolism 144, 155557. doi: 10.1016/j.metabol.2023.155557 37080353

[B34] Katelaris P. Hunt R. Bazzoli F. Cohen H. Fock K. M. Gemilyan M.. (2023). Helicobacter pylori world gastroenterology organization global guideline. J. Clin. Gastroenterol. 57, 111–126. doi: 10.1097/MCG.0000000000001719 36598803

[B35] Kohara K. Ochi M. Tabara Y. Nagai T. Igase M. Miki T. (2011). Leptin in sarcopenic visceral obesity: possible link between adipocytes and myocytes. PloS One 6, e24633. doi: 10.1371/journal.pone.0024633 21931785 PMC3170390

[B36] Lemieux I. Després J.-P. (2020). Metabolic syndrome: past, present and future. Nutrients 12, 3501. doi: 10.3390/nu12113501 33202550 PMC7696383

[B37] Lin Y.-L. Wang C.-H. Lai Y.-H. Kuo C.-H. Syu R.-J. Hsu B.-G. (2018). Negative correlation between leptin serum levels and sarcopenia in hemodialysis patients. Int. J. Clin. Exp. Pathol. 11, 1715–1723. 31938275 PMC6958142

[B38] Linz B. Balloux F. Moodley Y. Manica A. Liu H. Roumagnac P.. (2007). An African origin for the intimate association between humans and Helicobacter pylori. Nature 445, 915–918. doi: 10.1038/nature05562 17287725 PMC1847463

[B39] Lj F. Mo H.-L. (2017)Sarcopenia and the new ICD-10-CM code: screening, staging, and diagnosis considerations. In: Federal practitioner: for the health care professionals of the VA, DoD, and PHS. (Accessed November 22, 2023). PMC5576154 28867927

[B40] Logan R. P. (1998). Urea breath tests in the management of Helicobacter pylori infection. Gut 43 Suppl 1, S47–S50. doi: 10.1136/gut.43.2008.s47 9764040 PMC1766595

[B41] Londhe P. Guttridge D. C. (2015). Inflammation induced loss of skeletal muscle. Bone 80, 131–142. doi: 10.1016/j.bone.2015.03.015 26453502 PMC4600538

[B42] Malfertheiner P. Megraud F. O’Morain C. A. Gisbert J. P. Kuipers E. J. Axon A. T.. (2017). Management of Helicobacter pylori infection-the Maastricht V/Florence Consensus Report. Gut 66, 6–30. doi: 10.1136/gutjnl-2016-312288 27707777

[B43] Marshall B. J. Warren J. R. (1984). Unidentified curved bacilli in the stomach of patients with gastritis and peptic ulceration. Lancet 1, 1311–1315. doi: 10.1016/s0140-6736(84)91816-6 6145023

[B44] Meng W.-P. Wang Z.-Q. Deng J.-Q. Liu Y. Deng M.-M. Lü M.-H. (2016). The role of H. pylori cagA in regulating hormones of functional dyspepsia patients. Gastroenterol. Res. Pract. 2016, 7150959. doi: 10.1155/2016/7150959 27840636 PMC5093276

[B45] Moon S.-S. (2014). Low skeletal muscle mass is associated with insulin resistance, diabetes, and metabolic syndrome in the Korean population: the Korea National Health and Nutrition Examination Survey (KNHANES) 2009-2010. Endocr. J. 61, 61–70. doi: 10.1507/endocrj.ej13-0244 24088600

[B46] Nishikawa H. Fukunishi S. Asai A. Yokohama K. Ohama H. Nishiguchi S.. (2021). Sarcopenia, frailty and type 2 diabetes mellitus (Review). Mol. Med. Rep. 24, 854. doi: 10.3892/mmr.2021.12494 34651658

[B47] Nocon M. Kuhlmann A. Leodolter A. Roll S. Vauth C. Willich S. N.. (2009). Efficacy and cost-effectiveness of the 13C-urea breath test as the primary diagnostic investigation for the detection of Helicobacter pylori infection compared to invasive and non-invasive diagnostic tests. GMS Health Technol. Assess. 5, Doc14. doi: 10.3205/hta000076 21289901 PMC3011289

[B48] Ohtani M. Ge Z. García A. Rogers A. B. Muthupalani S. Taylor N. S.. (2011). 17 β-estradiol suppresses Helicobacter pylori-induced gastric pathology in male hypergastrinemic INS-GAS mice. Carcinogenesis 32, 1244–1250. doi: 10.1093/carcin/bgr072 21565825 PMC3149202

[B49] PapadopoulouS. K.TsintavisP.PotsakiP.PapandreouD. (2020). Differences in the prevalence of sarcopenia in community-dwelling, nursing home and hospitalized individuals. A systematic review and meta-analysis. J. Nutr. Health Aging 24, 83–90. doi: 10.1007/s12603-019-1267-x 31886813

[B50] Petermann-Rocha F. Balntzi V. Gray S. R. Lara J. Ho F. K. Pell J. P.. (2022). Global prevalence of sarcopenia and severe sarcopenia: a systematic review and meta-analysis. J. cachexia sarcopenia Muscle 13, 86–99. doi: 10.1002/jcsm.12783 34816624 PMC8818604

[B51] Ricci C. Holton J. Vaira D. (2007). Diagnosis of Helicobacter pylori: invasive and non-invasive tests. Best Pract. Res. Clin. Gastroenterol. 21, 299–313. doi: 10.1016/j.bpg.2006.11.002 17382278

[B52] Rubio-Ruiz M. E. Guarner-Lans V. Pérez-Torres I. Soto M. E. (2019). Mechanisms underlying metabolic syndrome-related sarcopenia and possible therapeutic measures. Int. J. Mol. Sci. 20, 647. doi: 10.3390/ijms20030647 30717377 PMC6387003

[B53] Runolfsdottir H. L. Sigurdsson G. Franzson L. Indridason O. S. (2015). Gender comparison of factors associated with age-related differences in bone mineral density. Arch. Osteoporos 10, 214. doi: 10.1007/s11657-015-0214-7 26239743

[B54] Ryall J. G. Schertzer J. D. Lynch G. S. (2008). Cellular and molecular mechanisms underlying age-related skeletal muscle wasting and weakness. Biogerontology 9, 213–228. doi: 10.1007/s10522-008-9131-0 18299960

[B55] Saklayen M. G. (2018). The global epidemic of the metabolic syndrome. Curr. Hypertens. Rep. 20, 12. doi: 10.1007/s11906-018-0812-z 29480368 PMC5866840

[B56] Sanada K. Iemitsu M. Murakami H. Gando Y. Kawano H. Kawakami R.. (2012). Adverse effects of coexistence of sarcopenia and metabolic syndrome in Japanese women. Eur. J. Clin. Nutr. 66, 1093–1098. doi: 10.1038/ejcn.2012.43 22569087

[B57] Serra-PratM.PapiolM.MonteisR.PalomeraE.CabréM. (2015). Relationship between plasma ghrelin levels and sarcopenia in elderly subjects: A cross-sectional study. J. Nutr. Health Aging 19, 669–672. doi: 10.1007/s12603-015-0550-8 26054503

[B58] Sk P. (2020). Sarcopenia: A contemporary health problem among older adult populations. Nutrients 12, 1293. doi: 10.3390/nu12051293 32370051 PMC7282252

[B59] Walowski C. O. Braun W. Maisch M. J. Jensen B. Peine S. Norman K.. (2020). Reference values for skeletal muscle mass - current concepts and methodological considerations. Nutrients 12, 755. doi: 10.3390/nu12030755 32178373 PMC7146130

[B60] Wang J. Leung K.-S. Chow S. K.-H. Cheung W.-H. (2017). Inflammation and age-associated skeletal muscle deterioration (sarcopaenia). J. Orthop Translat 10, 94–101. doi: 10.1016/j.jot.2017.05.006 29662761 PMC5822997

[B61] Watanabe T. Asano N. Fichtner-Gorelick P. L. Tsuji Y. Matsumoto Y.. (2010). NOD1 contributes to mouse host defense against Helicobacter pylori *via* induction of type I IFN and activation of the ISGF3 signaling pathway. J. Clin. Invest. 120, 1645–1662. doi: 10.1172/JCI39481 20389019 PMC2860924

[B62] Wu S.-E. Chen W.-L. (2021). Detrimental relevance of Helicobacter pylori infection with sarcopenia. Gut Pathog. 13, 67. doi: 10.1186/s13099-021-00464-y 34782007 PMC8591825

[B63] Yang L. Smith L. Hamer M. (2019). Gender-specific risk factors for incident sarcopenia: 8-year follow-up of the English longitudinal study of ageing. J. Epidemiol. Community Health 73, 86–88. doi: 10.1136/jech-2018-211258 30368480

[B64] Zamani M. Ebrahimtabar F. Zamani V. Miller W. H. Alizadeh-Navaei R. Shokri-Shirvani J.. (2018). Systematic review with meta-analysis: the worldwide prevalence of Helicobacter pylori infection. Aliment Pharmacol. Ther. 47, 868–876. doi: 10.1111/apt.14561 29430669

[B65] Zhang J.-X. Li J. Chen C. Yin T. Wang Q.-A. Li X.-X.. (2021). Reference values of skeletal muscle mass, fat mass and fat-to-muscle ratio for rural middle age and older adults in western China. Arch. Gerontol Geriatr. 95, 104389. doi: 10.1016/j.archger.2021.104389 33713879

